# *rac*-(2R*,3S*,5S*,6R*,7S*,8S*)-7,8-Dichloro­bicyclo­[2.2.2]octane-2,3,5,6-tetrayl tetra­acetate

**DOI:** 10.1107/S160053680900484X

**Published:** 2009-02-13

**Authors:** Ertan Şahin, Arif Baran, Metin Balcı

**Affiliations:** a Department of Chemistry, Faculty of Science, Atatürk University, 25240 Erzurum, Turkey; b Department of Chemistry, Faculty of Science, Sakarya University, 54100 Sakarya, Turkey; c Department of Chemistry, Faculty of Science, Middle East Technical University, 06531 Ankara, Turkey

## Abstract

The title compound, C_16_H_20_Cl_2_O_8_, contains a central bicyclo­[2.2.2]octane skeleton with slightly twisted conformation. In this structure, the C—C bond lengths are in the range 1.525 (2)–1.552 (2) Å. Two sides of this skeleton have *cis*,*cis* acet­oxy substituents and the Cl atoms have a *trans* arrangement. An extensive network of weak C—H⋯O interactions stabilizes the crystal structure.

## Related literature

For background information on inositol and its derivatives, see: Michell (2008 ▶); Reitz (1991 ▶); Dwek (1996 ▶); Billington *et al.* (1994 ▶); Varki (1993 ▶); Heightman & Vasella (1991 ▶). For background on the carba-analogues of oligosaccharides, see: Ogawa *et al.* (2000 ▶, 1988 ▶); Saumi (1990 ▶); Saumi & Ogawa (1990 ▶). For related structures, see: Baran *et al.* (2008 ▶); Mehta *et al.* (2007 ▶); Shih *et al.* (2007 ▶); Gültekin *et al.* (2004 ▶); Mehta & Ramesh (2001 ▶); Balcı (1997 ▶); Balcı et al. (1990 ▶);Ülkü *et al.* (1995 ▶); Buser & Vasella (2006 ▶).
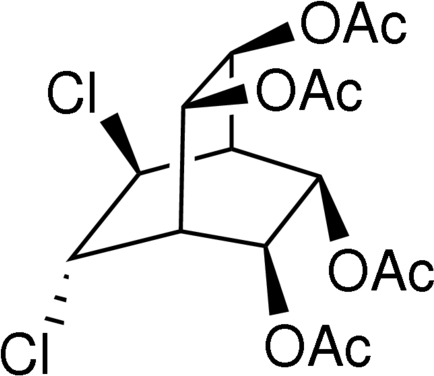


## Experimental

### 

#### Crystal data

C_16_H_20_Cl_2_O_8_
*M* *_r_* = 411.22
Monoclinic, 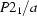

*a* = 10.1061 (3) Å
*b* = 13.3383 (4) Å
*c* = 14.2229 (3) Å
β = 90.189 (2)°
*V* = 1917.21 (9) Å^3^
*Z* = 4
Mo *K*α radiation
μ = 0.38 mm^−1^
*T* = 294 K
0.5 × 0.3 × 0.2 mm

#### Data collection

Rigaku R-AXIS RAPID-S diffractometer
Absorption correction: multi-scan (Blessing, 1995 ▶) *T*_min_ = 0.873, *T*_max_ = 0.927
54750 measured reflections
5628 independent reflections
5575 reflections with *I* > 2σ(*I*)
*R*_int_ = 0.024

#### Refinement

*R*[*F* ^2^ > 2σ(*F* ^2^)] = 0.065
*wR*(*F* ^2^) = 0.157
*S* = 1.32
5628 reflections
239 parameters
H-atom parameters constrained
Δρ_max_ = 0.38 e Å^−3^
Δρ_min_ = −0.38 e Å^−3^

### 

Data collection: *CrystalClear* (Rigaku/MSC, 2005 ▶); cell refinement: *CrystalClear*; data reduction: *CrystalClear*; program(s) used to solve structure: *SHELXS97* (Sheldrick, 2008 ▶); program(s) used to refine structure: *SHELXL97* (Sheldrick, 2008 ▶); molecular graphics: *ORTEP-3 for Windows* (Farrugia, 1997 ▶); software used to prepare material for publication: *WinGX* (Farrugia, 1999 ▶).

## Supplementary Material

Crystal structure: contains datablocks global, I. DOI: 10.1107/S160053680900484X/kp2205sup1.cif

Structure factors: contains datablocks I. DOI: 10.1107/S160053680900484X/kp2205Isup2.hkl

Additional supplementary materials:  crystallographic information; 3D view; checkCIF report

## Figures and Tables

**Table 1:** Hydrogen-bond geometry (Å, °)

*D*—H⋯*A*	*D*—H	H⋯*A*	*D*⋯*A*	*D*—H⋯*A*
C7—H7⋯O6^i^	0.98	2.46	3.425 (3)	169
C8—H8⋯O8^ii^	0.98	2.42	3.377 (3)	166
C16—H16*A*⋯O7^ii^	0.96	2.53	3.416 (4)	154
C12—H12*B*⋯O5^iii^	0.96	2.52	3.297 (4)	137
C6—H6⋯O7^iv^	0.98	2.60	3.240 (3)	123

Symmetry codes: (i) 
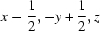
; (ii) 
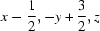
; (iii) 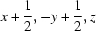; (iv) 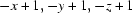.

**Fig. 1.**
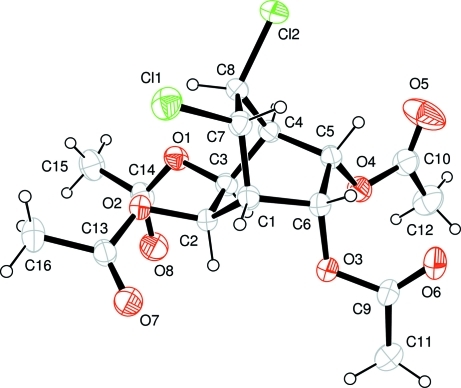
An ORTEP-3 (Farrugia, 1997) drawing of the title molecule with the atom-numbering scheme. The displacement ellipsoids are drawn at the 50% probability level.

**Fig. 2.**
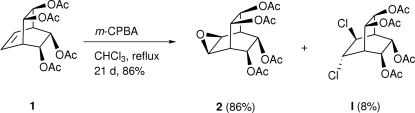
The formation of the title compound.

## supplementary crystallographic information

### Comment

Glycosidases and their inhibitors have been the subject of much research in the last decade. Inositols (cyclohexanehexols) are sugar-like molecules. There are nine stereoisomers, all of which may be referred to as inositol (Michell, 2008; Reitz, 1991). The most prominent naturally occurring form is myo-inositol, *cis*-1,2,3,5-*trans*-4,6-cyclohexanehexol and it is actively involved in cellular events and processes. Inositol and their derivatives can inhibit the glycosidases and affect many biological processes (Dwek, 1996; Billington *et al.* 1994; Varki, 1993; Heightman & Vasella 1991). Carba-analogues of oligosaccharides (carbasugar) generated by replacing the endocyclic oxygen atom in monosaccharides (Ogawa *et al.* 2000 and 1988; Saumi, 1990; Saumi & Ogawa, 1990) are thought to be more potent drug candidates than natural sugars, since they are hydrolytically stable.

New synthetic methodologies for various inositols and their derivatives have been developed. After this discovery, an enormous increase in the synthesis of cyclitol derivatives (Mehta *et al.* 2007; Shih *et al.* 2007; Gültekin *et al.* 2004; Mehta & Ramesh, 2001; Balcı, 1997; Balcı *et al.*1990) was observed since these show glycosidase inhibitory properties. More recently, a bridged and bicyclic system, the racemic *gluco*-configured norbornane has been synthesised and tested as inhibitor of β-glycosidases (Buser & Vasella, 2006). They noticed that the configuration of the hydroxy group play an important role in inhibitor activity. Motivated by the medical value of certain cyclitol derivatives, we were interested in designing a new generation of possible glycosidase inhibitors with the bicyclic structures having bicyclo[2.2.2]octane skeleton.

For the construction of the bicyclo[2.2.2]octane skeleton we started from 2,2-dimethyl-3a,7a-dihydro-1,3-benzo-dioxole and vinylene carbonate and synthesized the tetraacetate 1 in 3 steps (Baran *et al.*, 2008).

For the synthesis of isomeric hexols with bicyclo[2.2.2]octane skeleton, the tetracetate 1 was reacted with *m*-CPBA. The reaction was completed after 21 days by refluxing in chloroform. Recently, we isolated a side product I in 8% yields beside the major product 2 (86%). The structure of the side product was confirmed by NMR-spectroscopic studies. The incorporation of the chlorine atoms into the molecule and their configuration were determined by X-ray diffraction analysis. The title compound (I) C_16_H_20_O_8_Cl_2_ contains a central bicyclo[2.2.2]octane skeleton with slightly twisted conformation. In this structure C—C bond lengths are in the range of 1.525 (2)- 1.552 (2) Å. Two sides of this skeleton have *cis*, *cis* –OAc substituents. In addition to this, Cl atoms have *trans* stereochemistry at the other side (C7—Cl1=1.796 (2), C8—Cl2=1.798 (2) Å). Intermolecular C—H···O hydrogen bonds are effective in determining the molecular conformation and the crystal structure of the title compound (Table 1).

### Experimental

Oxidation of 2S,3R,5R,6S-rel-Bicyclo[2.2.2]oct-7-ene-2,3,5,6-tetraacetate with m-CPBA in chloroform was performed as followed. (1.0 g, 2.94 mmol) tetraacetate 1 in 100 ml of chloroform was reacted with (1.50 g, 6 mmol, 70%) m-CPBA as described in the literature (Baran et al., 2008). Evaporation of solvent under reduced pressure and recrystallization of product from ethyl acetate gave epoxide 2. After separation of epoxide 2, the solvent was removed and the residue was dissolved in ether and crystallization at 273 K gave colourless crystals of the dichloro compound I (97 mg, 8%) m.p. 469–472 K. ^1^H NMR (400 MHz, CDCl_3_) δ 5.41 (dd, J = 8.0 and 4.4 Hz, 1H), 5.33–5.28 (m, 3H), 4.59 (dd, J = 7.6 and 2. 0 Hz, 1H), 3.97 (br d, J = 7.6 Hz, 1H), 2.92 (dt, J= 4.4 and 1.2 and Hz, 1H), 2.43 (q, J = 2.0 Hz, 1H), 2.14 (s, –CH_3_), 2.11 (s,-CH_3_), 2.074 (s, –CH_3_), 2.07 (s, –CH_3_). ^13^C NMR (100 MHz, CDCl_3_) δ 168.1, 167.8, 167.6, 167.4, 64.7, 63.6, 62.3, 61.9, 57.8, 56.5, 43.9, 40.2, 19.2, 18.8, 18.7, 18.6. IR (KBr, cm^-1^) 2984, 2968, 1758, 1431, 1370, 1201, 982, 900. Anal. Calcd for C_16_H_20_C_l2_O_8_: C, 46.73; H, 4.90. Found: C, 46.80; H, 5.16.

Chlorination of 2S*,3R*,5R*,6S*--Bicyclo[2.2.2]oct-7-ene-2,3,5,6-tetraacetate was according the described procedure. (0.3 g, 0.88 mmol) tetraacetate 1 was dissolved in 100 ml of dichloromethane. Chlorine gas (generated from the reaction of KMnO_4_ with HCl) was passed through the solution. After 20 m the gas flow was stopped and the flask was closed with a stopper. The mixture was stirred at room temperature for 3 h, and then the solvent was evaporated. The crude product was dissolved in ether and crystallized at 273 K to give (0.29 g, 80%) colourless crystals of I (m.p.469–472 K). The interesting feature of this reaction is the unusual formations of chlorine adduct I during epoxidation reaction. A similar reaction was also observed during the epoxidation reaction of a benzobarrelene derivative (Ülkü et al., 1995) which was also completed in three weeks. We assume that solvent, chloroform undergoes a slow oxidation reaction with the m-chloroperbenzoic acid and generates chlorine which adds to the double bond.

To test whether the adduct I has been generated by addition of free chlorine to the double bond or by other mechanism, we treated the tetraacetate 1 with chlorine gas for 3 h. The chlorine added to the double bond in 1 in a yield of 80% and gave I, which was identical with the adduct isolated from the epoxidation reaction of 1 as the side product.

To the best of our knowledge, chlorine addition to a double bond during an epoxidation reaction has been not presented in the literature. This reaction can be probably encountered only during those reactions which are completed in 2–3 weeks because of the very slow oxidation of chloroform.

### Refinement

H atoms were placed in geometrically idealized positions (C—H=0.96–0.98 Å) and treated as riding, with *U*_iso_(H)=1.2*U*_eq_(C)(for methine) or 1.5*U*_eq_(methyl C).

### Crystal data

**Table:**

C_16_H_20_Cl_2_O_8_	*F*(000) = 856
*M**_r_* = 411.22	*D*_x_ = 1.425 Mg m^−^^3^
Monoclinic, *P*2_1_/*a*	Mo *K*α radiation, λ = 0.71073 Å
Hall symbol: -P 2yab	Cell parameters from 16740 reflections
*a* = 10.1061 (3) Å	θ = 2.9–30.0°
*b* = 13.3383 (4) Å	µ = 0.38 mm^−^^1^
*c* = 14.2229 (3) Å	*T* = 294 K
β = 90.189 (2)°	Block, colourless
*V* = 1917.21 (9) Å^3^	0.5 × 0.3 × 0.2 mm
*Z* = 4	

### Data collection

**Table:**

Rigaku R-AXIS RAPID-S diffractometer	5628 independent reflections
graphite	5575 reflections with *I* > 2σ(*I*)
Detector resolution: 10 pixels mm^-1^	*R*_int_ = 0.024
dtprofit.ref scans	θ_max_ = 30.2°, θ_min_ = 2.9°
Absorption correction: multi-scan (Blessing, 1995)	*h* = −14→14
*T*_min_ = 0.873, *T*_max_ = 0.927	*k* = −18→18
54750 measured reflections	*l* = −20→20

### Refinement

**Table:**

Refinement on *F*^2^	Primary atom site location: structure-invariant direct methods
Least-squares matrix: full	Secondary atom site location: difference Fourier map
*R*[*F*^2^ > 2σ(*F*^2^)] = 0.065	Hydrogen site location: inferred from neighbouring sites
*wR*(*F*^2^) = 0.157	H-atom parameters constrained
*S* = 1.32	*w* = 1/[σ^2^(*F*_o_^2^) + (0.0494*P*)^2^ + 0.7822*P*] where *P* = (*F*_o_^2^ + 2*F*_c_^2^)/3
5628 reflections	(Δ/σ)_max_ < 0.001
239 parameters	Δρ_max_ = 0.38 e Å^−^^3^
0 restraints	Δρ_min_ = −0.38 e Å^−^^3^

### Special details

**Table:**

Geometry. All s.u.'s (except the s.u. in the dihedral angle between two l.s. planes) are estimated using the full covariance matrix. The cell s.u.'s are taken into account individually in the estimation of s.u.'s in distances, angles and torsion angles; correlations between s.u.'s in cell parameters are only used when they are defined by crystal symmetry. An approximate (isotropic) treatment of cell s.u.'s is used for estimating s.u.'s involving l.s. planes.
Refinement. Refinement of *F*^2^ against ALL reflections. The weighted *R*-factor *wR* and goodness of fit *S* are based on *F*^2^, conventional *R*-factors *R* are based on *F*, with *F* set to zero for negative *F*^2^. The threshold expression of *F*^2^ > 2σ(*F*^2^) is used only for calculating *R*-factors(gt) *etc*. and is not relevant to the choice of reflections for refinement. *R*-factors based on *F*^2^ are statistically about twice as large as those based on *F*, and *R*- factors based on ALL data will be even larger.

### Fractional atomic coordinates and isotropic or equivalent isotropic displacement parameters (Å^2^)

**Table:**

	*x*	*y*	*z*	*U*_iso_*/*U*_eq_	
O1	0.43075 (15)	0.68316 (10)	0.83281 (11)	0.0493 (3)	
O2	0.40605 (14)	0.67994 (10)	0.65495 (10)	0.0456 (3)	
O3	0.65280 (15)	0.43371 (11)	0.67091 (11)	0.0515 (4)	
O4	0.61501 (15)	0.40594 (12)	0.84846 (12)	0.0562 (4)	
O6	0.7006 (2)	0.27009 (13)	0.68650 (16)	0.0760 (6)	
C1	0.4340 (2)	0.49728 (14)	0.64831 (14)	0.0432 (4)	
H1	0.4388	0.4942	0.5796	0.052*	
O7	0.5083 (2)	0.68542 (15)	0.51586 (12)	0.0707 (5)	
C3	0.49349 (19)	0.59673 (14)	0.79258 (14)	0.0431 (4)	
H3	0.5857	0.5931	0.8139	0.052*	
O5	0.5382 (3)	0.2886 (2)	0.94322 (18)	0.1158 (11)	
C9	0.7365 (2)	0.35432 (17)	0.67186 (17)	0.0565 (5)	
C5	0.49031 (19)	0.41067 (14)	0.79928 (14)	0.0445 (4)	
H5	0.4368	0.3518	0.8151	0.053*	
C8	0.27942 (19)	0.50853 (14)	0.78399 (14)	0.0436 (4)	
H8	0.2452	0.5772	0.7868	0.052*	
C2	0.48878 (19)	0.59749 (13)	0.68357 (14)	0.0411 (4)	
H2	0.5783	0.6073	0.6589	0.049*	
C7	0.2918 (2)	0.47717 (15)	0.68102 (14)	0.0451 (4)	
H7	0.2761	0.4048	0.6773	0.054*	
C6	0.5167 (2)	0.41264 (14)	0.69188 (15)	0.0454 (4)	
H6	0.4911	0.3483	0.6640	0.055*	
C13	0.4288 (2)	0.71914 (16)	0.56923 (15)	0.0492 (4)	
C4	0.41742 (19)	0.50589 (14)	0.82877 (14)	0.0420 (4)	
H4	0.4103	0.5088	0.8974	0.050*	
C14	0.4989 (2)	0.76967 (16)	0.82909 (17)	0.0551 (5)	
C12	0.7623 (3)	0.3419 (3)	0.9605 (2)	0.0785 (8)	
H12C	0.7600	0.3159	1.0234	0.118*	
H12A	0.7942	0.4097	0.9617	0.118*	
H12B	0.8202	0.3015	0.9229	0.118*	
C15	0.4182 (3)	0.85465 (19)	0.8642 (2)	0.0736 (7)	
H15A	0.4056	0.8478	0.9307	0.110*	
H15B	0.3337	0.8546	0.8331	0.110*	
H15C	0.4630	0.9166	0.8514	0.110*	
C10	0.6262 (3)	0.3398 (2)	0.91940 (16)	0.0590 (5)	
C11	0.8745 (3)	0.3867 (2)	0.6533 (2)	0.0790 (8)	
H11C	0.9172	0.4033	0.7116	0.119*	
H11A	0.8737	0.4444	0.6130	0.119*	
H11B	0.9218	0.3332	0.6232	0.119*	
C16	0.3424 (3)	0.8084 (2)	0.5524 (2)	0.0740 (8)	
H16C	0.3678	0.8405	0.4948	0.111*	
H16B	0.3521	0.8548	0.6036	0.111*	
H16A	0.2518	0.7873	0.5481	0.111*	
O8	0.60945 (19)	0.77413 (14)	0.79959 (18)	0.0809 (6)	
Cl2	0.16803 (6)	0.42687 (5)	0.84582 (5)	0.06100 (17)	
Cl1	0.16992 (6)	0.53836 (5)	0.60893 (5)	0.06395 (18)	

### Atomic displacement parameters (Å^2^)

**Table:**

	*U*^11^	*U*^22^	*U*^33^	*U*^12^	*U*^13^	*U*^23^
O1	0.0548 (8)	0.0384 (7)	0.0547 (8)	−0.0079 (6)	0.0039 (6)	−0.0063 (6)
O2	0.0461 (7)	0.0389 (7)	0.0519 (7)	0.0066 (5)	0.0045 (6)	0.0054 (5)
O3	0.0473 (8)	0.0395 (7)	0.0676 (9)	0.0078 (6)	0.0118 (7)	0.0062 (6)
O4	0.0438 (8)	0.0582 (9)	0.0665 (9)	0.0014 (6)	−0.0081 (7)	0.0147 (7)
O6	0.0746 (12)	0.0437 (9)	0.1097 (16)	0.0146 (8)	0.0097 (11)	0.0094 (9)
C1	0.0472 (10)	0.0381 (9)	0.0445 (9)	0.0027 (7)	0.0024 (7)	0.0014 (7)
O7	0.0774 (12)	0.0783 (12)	0.0564 (10)	0.0201 (10)	0.0151 (9)	0.0154 (9)
C3	0.0414 (9)	0.0386 (9)	0.0493 (10)	−0.0036 (7)	−0.0006 (7)	0.0007 (7)
O5	0.0850 (15)	0.163 (3)	0.0992 (17)	−0.0404 (16)	−0.0287 (12)	0.0788 (18)
C9	0.0581 (12)	0.0464 (11)	0.0650 (13)	0.0160 (9)	0.0092 (10)	0.0055 (10)
C5	0.0413 (9)	0.0396 (9)	0.0527 (10)	0.0002 (7)	−0.0017 (8)	0.0076 (8)
C8	0.0406 (9)	0.0368 (9)	0.0534 (10)	−0.0030 (7)	0.0038 (8)	0.0028 (7)
C2	0.0382 (8)	0.0356 (8)	0.0496 (10)	0.0026 (7)	0.0038 (7)	0.0056 (7)
C7	0.0446 (10)	0.0382 (9)	0.0525 (10)	−0.0019 (7)	−0.0041 (8)	−0.0013 (8)
C6	0.0449 (10)	0.0358 (9)	0.0556 (11)	0.0020 (7)	0.0042 (8)	0.0014 (8)
C13	0.0514 (11)	0.0437 (10)	0.0525 (11)	0.0014 (8)	−0.0026 (9)	0.0080 (8)
C4	0.0425 (9)	0.0396 (9)	0.0439 (9)	−0.0033 (7)	0.0010 (7)	0.0031 (7)
C14	0.0606 (13)	0.0411 (10)	0.0636 (13)	−0.0115 (9)	−0.0101 (10)	0.0013 (9)
C12	0.0621 (15)	0.104 (2)	0.0691 (16)	0.0164 (15)	−0.0158 (13)	0.0076 (15)
C15	0.092 (2)	0.0414 (12)	0.0874 (19)	−0.0048 (12)	−0.0021 (15)	−0.0067 (12)
C10	0.0579 (13)	0.0691 (14)	0.0500 (11)	0.0075 (11)	−0.0047 (10)	0.0066 (10)
C11	0.0552 (14)	0.0768 (18)	0.105 (2)	0.0165 (13)	0.0163 (14)	0.0124 (16)
C16	0.0815 (18)	0.0573 (14)	0.0833 (18)	0.0202 (13)	−0.0038 (14)	0.0190 (13)
O8	0.0582 (11)	0.0543 (10)	0.1301 (18)	−0.0180 (8)	0.0015 (11)	0.0029 (11)
Cl2	0.0510 (3)	0.0560 (3)	0.0761 (4)	−0.0096 (2)	0.0142 (3)	0.0077 (3)
Cl1	0.0534 (3)	0.0690 (4)	0.0694 (4)	0.0004 (3)	−0.0176 (3)	0.0018 (3)

### Geometric parameters (Å, °)

**Table:**

O1—C14	1.345 (2)	C8—Cl2	1.7982 (19)
O1—C3	1.436 (2)	C8—H8	0.9800
O2—C13	1.347 (2)	C2—H2	0.9800
O2—C2	1.439 (2)	C7—Cl1	1.796 (2)
O3—C9	1.355 (2)	C7—H7	0.9800
O3—C6	1.436 (2)	C6—H6	0.9800
O4—C10	1.345 (3)	C13—C16	1.495 (3)
O4—C5	1.441 (2)	C4—H4	0.9800
O6—C9	1.199 (3)	C14—O8	1.197 (3)
C1—C2	1.531 (3)	C14—C15	1.484 (4)
C1—C6	1.534 (3)	C12—C10	1.493 (4)
C1—C7	1.536 (3)	C12—H12C	0.9600
C1—H1	0.9800	C12—H12A	0.9600
O7—C13	1.195 (3)	C12—H12B	0.9600
C3—C4	1.525 (3)	C15—H15A	0.9600
C3—C2	1.551 (3)	C15—H15B	0.9600
C3—H3	0.9800	C15—H15C	0.9600
O5—C10	1.173 (3)	C11—H11C	0.9600
C9—C11	1.484 (4)	C11—H11A	0.9600
C5—C4	1.528 (3)	C11—H11B	0.9600
C5—C6	1.552 (3)	C16—H16C	0.9600
C5—H5	0.9800	C16—H16B	0.9600
C8—C7	1.529 (3)	C16—H16A	0.9600
C8—C4	1.532 (3)		
			
C14—O1—C3	116.52 (17)	O3—C6—C5	112.04 (17)
C13—O2—C2	116.86 (15)	C1—C6—C5	108.37 (16)
C9—O3—C6	116.31 (17)	O3—C6—H6	109.8
C10—O4—C5	117.68 (18)	C1—C6—H6	109.8
C2—C1—C6	108.32 (16)	C5—C6—H6	109.8
C2—C1—C7	113.02 (16)	O7—C13—O2	123.09 (19)
C6—C1—C7	104.95 (16)	O7—C13—C16	126.3 (2)
C2—C1—H1	110.1	O2—C13—C16	110.6 (2)
C6—C1—H1	110.1	C3—C4—C5	108.89 (16)
C7—C1—H1	110.1	C3—C4—C8	107.50 (15)
O1—C3—C4	106.24 (15)	C5—C4—C8	110.13 (16)
O1—C3—C2	112.40 (15)	C3—C4—H4	110.1
C4—C3—C2	109.19 (15)	C5—C4—H4	110.1
O1—C3—H3	109.6	C8—C4—H4	110.1
C4—C3—H3	109.6	O8—C14—O1	122.4 (2)
C2—C3—H3	109.6	O8—C14—C15	126.5 (2)
O6—C9—O3	123.0 (2)	O1—C14—C15	111.1 (2)
O6—C9—C11	126.0 (2)	C10—C12—H12C	109.5
O3—C9—C11	111.0 (2)	C10—C12—H12A	109.5
O4—C5—C4	108.96 (17)	H12C—C12—H12A	109.5
O4—C5—C6	109.03 (16)	C10—C12—H12B	109.5
C4—C5—C6	109.92 (15)	H12C—C12—H12B	109.5
O4—C5—H5	109.6	H12A—C12—H12B	109.5
C4—C5—H5	109.6	C14—C15—H15A	109.5
C6—C5—H5	109.6	C14—C15—H15B	109.5
C7—C8—C4	108.35 (16)	H15A—C15—H15B	109.5
C7—C8—Cl2	110.86 (13)	C14—C15—H15C	109.5
C4—C8—Cl2	110.69 (13)	H15A—C15—H15C	109.5
C7—C8—H8	109.0	H15B—C15—H15C	109.5
C4—C8—H8	109.0	O5—C10—O4	122.5 (2)
Cl2—C8—H8	109.0	O5—C10—C12	126.6 (3)
O2—C2—C1	111.44 (16)	O4—C10—C12	110.9 (2)
O2—C2—C3	107.67 (15)	C9—C11—H11C	109.5
C1—C2—C3	109.37 (15)	C9—C11—H11A	109.5
O2—C2—H2	109.4	H11C—C11—H11A	109.5
C1—C2—H2	109.4	C9—C11—H11B	109.5
C3—C2—H2	109.4	H11C—C11—H11B	109.5
C8—C7—C1	108.79 (16)	H11A—C11—H11B	109.5
C8—C7—Cl1	111.38 (14)	C13—C16—H16C	109.5
C1—C7—Cl1	112.85 (14)	C13—C16—H16B	109.5
C8—C7—H7	107.9	H16C—C16—H16B	109.5
C1—C7—H7	107.9	C13—C16—H16A	109.5
Cl1—C7—H7	107.9	H16C—C16—H16A	109.5
O3—C6—C1	107.03 (15)	H16B—C16—H16A	109.5
			
C14—O1—C3—C4	164.71 (17)	C2—C1—C6—O3	−53.5 (2)
C14—O1—C3—C2	−75.9 (2)	C7—C1—C6—O3	−174.44 (16)
C6—O3—C9—O6	2.1 (4)	C2—C1—C6—C5	67.6 (2)
C6—O3—C9—C11	−177.5 (2)	C7—C1—C6—C5	−53.4 (2)
C10—O4—C5—C4	105.8 (2)	O4—C5—C6—O3	−14.1 (2)
C10—O4—C5—C6	−134.2 (2)	C4—C5—C6—O3	105.27 (18)
C13—O2—C2—C1	−85.8 (2)	O4—C5—C6—C1	−131.97 (17)
C13—O2—C2—C3	154.22 (17)	C4—C5—C6—C1	−12.6 (2)
C6—C1—C2—O2	−172.81 (15)	C2—O2—C13—O7	3.9 (3)
C7—C1—C2—O2	−57.0 (2)	C2—O2—C13—C16	−175.8 (2)
C6—C1—C2—C3	−53.9 (2)	O1—C3—C4—C5	−172.47 (15)
C7—C1—C2—C3	62.0 (2)	C2—C3—C4—C5	66.09 (19)
O1—C3—C2—O2	−7.5 (2)	O1—C3—C4—C8	68.23 (19)
C4—C3—C2—O2	110.12 (17)	C2—C3—C4—C8	−53.2 (2)
O1—C3—C2—C1	−128.73 (16)	O4—C5—C4—C3	67.1 (2)
C4—C3—C2—C1	−11.1 (2)	C6—C5—C4—C3	−52.3 (2)
C4—C8—C7—C1	−23.6 (2)	O4—C5—C4—C8	−175.22 (15)
Cl2—C8—C7—C1	−145.30 (14)	C6—C5—C4—C8	65.4 (2)
C4—C8—C7—Cl1	−148.64 (13)	C7—C8—C4—C3	74.40 (19)
Cl2—C8—C7—Cl1	89.70 (15)	Cl2—C8—C4—C3	−163.84 (13)
C2—C1—C7—C8	−41.9 (2)	C7—C8—C4—C5	−44.1 (2)
C6—C1—C7—C8	75.91 (19)	Cl2—C8—C4—C5	77.66 (18)
C2—C1—C7—Cl1	82.20 (18)	C3—O1—C14—O8	−4.8 (3)
C6—C1—C7—Cl1	−159.95 (14)	C3—O1—C14—C15	174.3 (2)
C9—O3—C6—C1	−156.87 (19)	C5—O4—C10—O5	−1.7 (4)
C9—O3—C6—C5	84.5 (2)	C5—O4—C10—C12	178.0 (2)

### Hydrogen-bond geometry (Å, °)

**Table:**

*D*—H···*A*	*D*—H	H···*A*	*D*···*A*	*D*—H···*A*
C7—H7···O6^i^	0.98	2.46	3.425 (3)	169
C8—H8···O8^ii^	0.98	2.42	3.377 (3)	166
C16—H16A···O7^ii^	0.96	2.53	3.416 (4)	154
C12—H12B···O5^iii^	0.96	2.52	3.297 (4)	137
C6—H6···O7^iv^	0.98	2.60	3.240 (3)	123

Symmetry codes: (i) *x*−1/2, −*y*+1/2, *z*; (ii) *x*−1/2, −*y*+3/2, *z*; (iii) *x*+1/2, −*y*+1/2, *z*; (iv) −*x*+1, −*y*+1, −*z*+1.
